# On a remarkable syndrome of cerebral atrophy associated with hyperammonaemia in childhood 

**DOI:** 10.1007/s10354-016-0492-8

**Published:** 2016-08-30

**Authors:** Andreas Rett

**Affiliations:** c/o M. Freilinger, Department of Paediatrics and Adolescent Medicine, Medical University of Vienna, Vienna, Austria

Regular monitoring and examination of more than 6000 brain-injured children over many years has more recently enabled us to identify a condition that shows strikingly consistent clinical, neurological and psychological symptoms among affected children, and defies classification as any known disorder discussed so far. Extensive studies have shown 22 girls to be affected with the syndrome.

These patients’ medical histories barely differ from the medical histories of other brain-injured children on our ward. Their genetic family history, medical history, pregnancies and delivery data are almost identical. One striking difference, however, is the higher rate of miscarriages and stillbirths prior to and after the patients’ births.

All children were born without signs of impairment. They developed normally during the first 9 months of early childhood, aside from a sucking weakness diagnosed in 30 % of cases.

However, delays in motor and mental development soon became evident: while they did learn to sit independently in good time and in a sufficient manner, their ability to stand was clearly retarded. Some children even learned to walk independently, although their gait would very soon be limited by apraxia. Numerous children acquired a few one- or two-syllable words, but stagnated in their language acquisition by the beginning of their third year of age.

Fig. 1 documents the clinical symptomatology and prevalence of individual steps of deterioration.

**Fig. 1:**
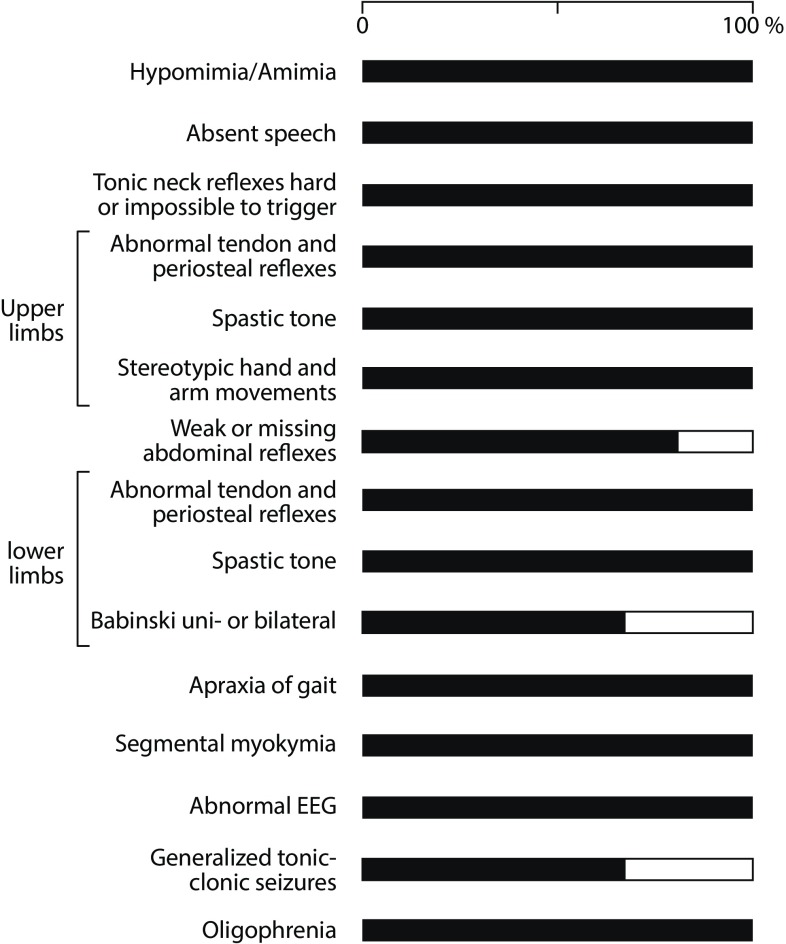
Graphic representation of the clinical symptomatology

Their facial expression shows little emotion, interspersed by spells of whining in some cases. Only strong acoustic or visual stimuli would trigger some sort of response. Their stare appears vacant, and they barely seem to recognise processes in their surroundings. Their cranial shape shows features of pseudo-microencephaly.

Utterances of speech are missing. Sometimes inarticulate sounds are produced in early stages of the disease.

The muscle tone in the upper limbs is spastically increased, depending on the stage and severity of disease, ranging from mild spasticity to a rigor-like tone. The tendon and periosteal reflexes are moderately hyperreflexive.

Stereotypic movements of hands and arms are the children’s most apparent symptom when observation commences. They are present almost constantly during the waking state and range from monotonously rhythmic elementary clapping or folding of hands; to hours-long rhythmic scratching, rubbing and knocking movements; to bizarre, rhythmic intertwining and pressing of fingers; and many variations thereof. External stimuli can intensify these movements significantly. Occasionally, these stereotypic movements of the upper limbs are accompanied by rocking movements and stereotypic forced breathing.

The muscle tone in the lower limbs is increased to a variable degree. The more advanced the disease the higher the tone, resulting in some cases in drop feet or, more frequently, in club feet.

The children’s gait—while they are still able to walk independently—can best be described as apraxic. Apparently their movement pattern, i. e. their movement planning, is impaired in spite of perfectly functional limbs. They walk slowly and rhythmically, take short steps and plant the whole soles of their feet on the ground, transferring the upper body from one leg to the other in wide, swaying movements, while keeping up the stereotypic movements of the upper limbs.

Where the tone is highly spastic and requires the children to use support, walking becomes more difficult, but the gait retains its characteristics.

The electroencephalogram (EEG) is diffusely altered, showing a distinct deterioration of spatial differentiation in the brain over the course of development. Epileptic seizures (grand mal) occurred in 15 of 22 children.

Psychological assessments showed all cases to be so strongly retarded that no quantifiable results could be obtained.

Due to the total loss of language as a means of expression and making contact, the loss of purposeful grasping, and because of the virtual inability to hold objects for short periods, the psychological examination confines itself to observation of sensory perception and motor skills in the broadest sense.

## Examinations

We believe that our biopsy findings are highly significant for evaluation of the condition.

A corticomedullary tissue sample showed different stages of diffusely atrophic nerve cell process with a reactive proliferation of astrocytes. These alterations are disease-specific and indicative of an endogenous degenerative cerebral atrophy.

Cytogenetic examinations showed normal female karyotypes.

Pneumoencephalographic examinations were unsuggestive of alterations in the subarachnoid spaces.

The electromyogram clearly showed a flexor spasticity in the lower limbs and highly atrophic quadriceps femoris muscles, which is suggestive of a damaged peripheral neuron.

Urine tests showed no pathological changes except for a significant increase in chlorides in the serum and liquor. However, there were no findings from blood tests or liver biopsies to suggest hepatic damage.

The already surprisingly consistent clinical picture was confirmed by a test of amino acids in the serum.

**Fig. 2:**
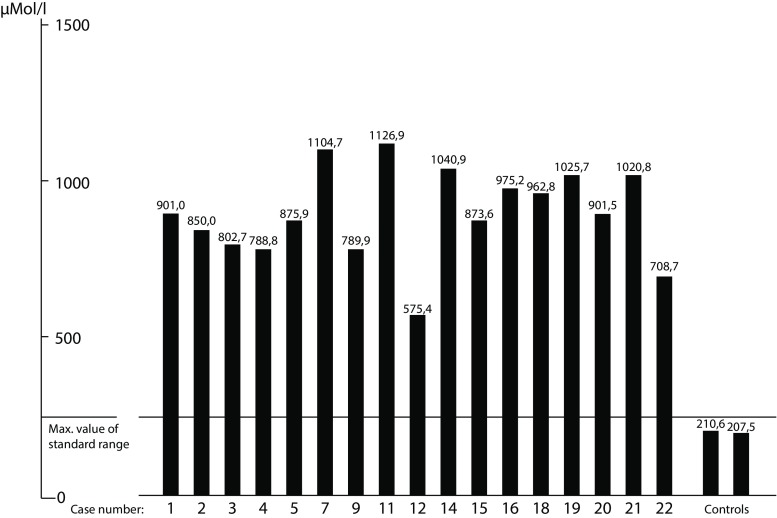
Values of NH_3 _in the serum

These tests were carried out in 17 of the 22 children associated with the syndrome (Fig. 2). The figure shows the ammonia levels to be up to 5‑times higher than normal. The other fractions were generally within normal limits.

This result appears to establish the pathogenesis of the disease, suggestive as it is that such a high concentration of ammonia has a toxic effect on neural tissue, as previously noted by Pavlov in the late 19^th^ century.

The fact that only the female gender has so far been affected by the disease has led us to believe that we are dealing with an inborn error of metabolism caused by a genetically determined disorder of urea synthesis.

Russel, Levin, Oberholzer and Sinclair were the first to report on hyperammonaemia in 1962. In the course of biochemical examinations, they found that a total cohort of four children with periodic phases of violent vomiting and subsequent lethargy or stupor showed a significant increase in levels of ammonia in the blood and liquor, accompanied by an elevated level of glutamine. These findings were confirmed in 1964 by the teams of Colombo, Richterich, Donath, Spahr and Rossi, and Freeman, Nicholson, Masland, Rowland and Carter.

Russel and colleagues went on to describe the resulting dementia and microcephaly in one of the children. The cited authors found that, in phases of vomiting, their patients were in a state similar to a hepatic coma. For our part, we neither observed such acutely dramatic phases nor recorded them by anamnesis.

One characteristic feature in our cohort of patients is the consistency of ammonia levels in the serum and liquor.

We have reason to believe that there is a certain correlation between the four cases mentioned above (all of whom were girls) and the 22 girls of our own cohort, although the latter no doubt showed a very specific progression.

The fact that the 22 children diagnosed with the present syndrome were paralleled by only eight cases of phenylketonuria shows that cerebral atrophy caused by hyperammonaemia is by no means a rare disease.

It seems imperative that hyperammonaemia be diagnosed or ruled out by means of a rapid and simple method as part of routine examinations of brain-injured children. Time will tell whether or not these results have the power to engender therapeutic measures. Our task now is to complete the clinical picture by carrying out detailed examinations of the enzymatic metabolism of these children as well as of their relatives.

## Conclusion

Monitoring more than 6000 brain-injured children with developmental disorders has enabled us over the years to identify a condition in 22 children (girls) characterised by strikingly consistent anamneses and clinical, neurological and psychological impairment patterns.

Apart from hyperammonaemia, typical clinical characteristics include:

1. Hypomimia/amimia,
2. Absent speech,
3. Stereotypic hand and arm movements,
4. Hyperreflexia,
5. Spastic hypertonia,
6. Apraxia of gait,
7. Epilepsy,
8. Extreme oligophrenia,
9. Gynaecotropism.

The condition becomes apparent by the end of the first year of life and progresses slowly. The oldest of the children we monitored is now 13 years old. All of them are girls. The EEG shows progressive loss of spatial differentiation in the brain. Cytogenetic examinations showed normal female karyotypes. The findings of pneumoencephalographic examinations were unsuspicious. Brain biopsies clearly revealed cerebral atrophy. Liver biopsies were unsuggestive of hepatic damage. The results of urine, blood and liquor tests were normal. Detailed tests of free amino acids in the serum were feasible for 17 children. All of these cases showed dramatically high ammonia levels, i. e. up to 5‑times higher than normal.

Given the toxic effect of ammonia on neural tissue, the diagnosed hyperammonaemia must be considered the cause of the atrophic process in the brain, although clinical findings also suggest some damage to the peripheral neuron.

The condition discussed is probably an inborn error of metabolism, more precisely a genetically determined disorder of urea synthesis.

Parallels with and fundamental differences to four cases of hyperammonaemia during violent bursts of vomiting reported by Russel et al., Freeman et al., and Colombo et al. are discussed above.

